# Supplementary material for: Association between oral microbiome and five types of respiratory infections: a two-sample Mendelian randomization study in east Asian population
Source: Front Microbiol. 2024 Apr 10;15:1392473. doi: 10.3389/fmicb.2024.1392473 (PMC11039966; doi:10.3389/fmicb.2024.1392473)
Supplement: Supplementary file 1 [file Data_Sheet_1.zip › Supplementary Materials/MR plots of saliva/Bronchitis/s__Rothia_mucilaginosa_B_mgs_3176/forest.pdf]

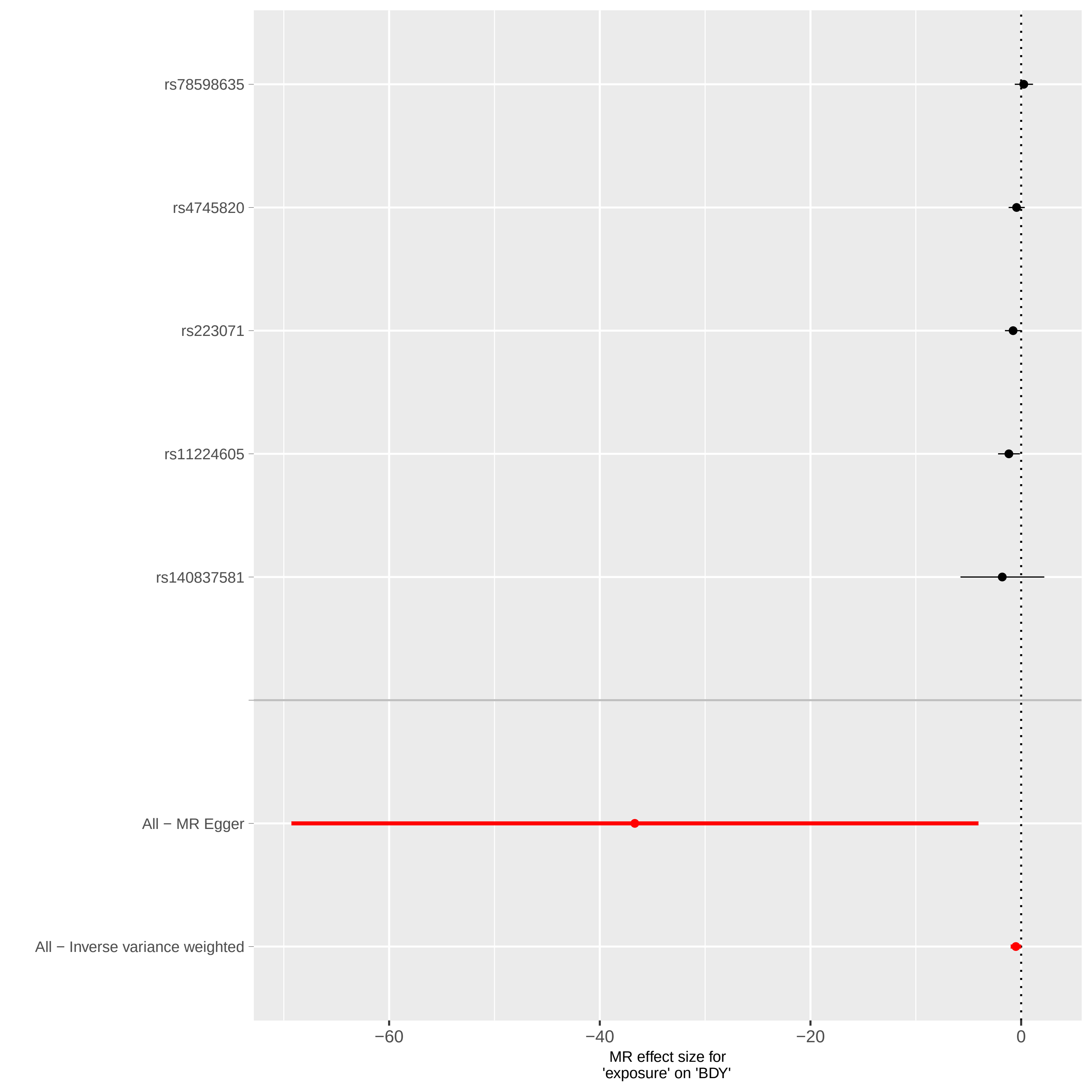
rs78598635
rs4745820
rs223071
rs11224605
rs140837581
All − MR Egger
All − Inverse variance weighted
−60
−40
−20
0
MR effect size for
'exposure' on 'BDY'
